# Arterial Stiffness and Hypertension in the Elderly

**DOI:** 10.3389/fcvm.2020.544302

**Published:** 2020-10-29

**Authors:** Stéphane Laurent, Pierre Boutouyrie

**Affiliations:** ^1^Assistance-Publique Hôpitaux de Paris, Université de Paris, Paris, France; ^2^PARCC-INSERM U970, Paris, France; ^3^Department of Pharmacology and Hôpital Européen Georges Pompidou, Paris, France

**Keywords:** age, arterial stiffness, cardiovascular events, central blood pressure, elderly, large artery, organ damage

## Abstract

Hypertension prevalence increases with age. Age and high blood pressure are the two main determinants of arterial stiffness. In elderly hypertensives, large arteries stiffen and systolic and pulse pressures increase, due to wave reflections. A major reason for measuring arterial stiffness in clinical practice in elderly hypertensive patients comes from the repeated demonstration that arterial stiffness and wave reflections have a predictive value for CV events. A large body of evidence has been published during the last two decades, concerning the epidemiology, pathophysiology, and pharmacology of large arteries in hypertension in various settings of age. Particularly, two expert consensus documents have reviewed the methodological agreements for measuring arterial stiffness. The concepts of Early Vascular Aging (EVA) and Supernormal Vascular Aging (SUPERNOVA) help to better understand on which determinants of arterial stiffness it is possible to act, in order to limit target organ damage and cardiovascular complications. This review will address the issues of the cellular and molecular mechanisms of arterial stiffening in elderly hypertensives, the consequences of arterial stiffening on central systolic and pulse (systolic *minus* diastolic, PP) pressures and target organs, the methodology for measuring arterial stiffness, central pulse pressure and wave reflection, the epidemiological determinants of arterial stiffening in elderly hypertensives, the pharmacology of arterial destiffening, and how the concepts of EVA and SUPERNOVA apply to the detection of organ damage and prevention of CV complications.

## Introduction

Hypertension prevalence increases with age. Age and high blood pressure are the two main determinants of arterial stiffness. In elderly hypertensives, large arteries stiffen and systolic and pulse pressures increase, due to wave reflections. There are two major reasons for measuring arterial stiffness in clinical practice in elderly hypertensive patients. Firstly the repeated demonstration that arterial stiffness and reflected waves are predictive for CV events (1–4). An important number of articles and reviews have been published during the last two decades, concerning various aspects of large arteries, including epidemiology, pathophysiology, and pharmacology. Particularly, two expert consensus documents have reviewed the methodological agreements for measuring arterial stiffness (1, 5). Secondly, arterial stiffness is a key factor for Early Vascular Aging (EVA) (6) and Supernormal Vascular Aging (SUPERNOVA) (7) which condition how the aging process will go on and helps to better understand on which determinants of arterial stiffness it is possible to act, in order to slow down target organ damage and cardiovascular complications usually associated with aging. This review will address the issues of the cellular and molecular mechanisms of arterial stiffening in elderly hypertensives, the consequences of arterial stiffening on central systolic and pulse (systolic *minus* diastolic, PP) pressures and target organs, the methodology for measuring arterial stiffness, central pulse pressure and wave reflection, the epidemiological determinants of arterial stiffening in elderly hypertensives, the pharmacology of arterial destiffening, and how the concepts of EVA and SUPERNOVA apply to the detection of organ damage and prevention of CV complications.

## Cellular and Molecular Determinants of Arterial Stiffness in the Elderly Hypertensive

Various mechanisms are involved in aortic stiffening, in response to age and cardiovascular risk factors. They include breaks in elastin fibers, cross-links of the elastin network, and accumulation of collagen. In addition, fibrosis, inflammation, medial smooth muscle necrosis, calcifications, and diffusion of macromolecules within the arterial wall can play a role (8–11). More specifically, two components are of major importance: the extracellular matrix (ECM) proteins which support the mechanical load, and the vascular smooth muscle cells (VSMCs) which regulate actin-myosin contraction and mediate mechanotransduction in cell-ECM homeostasis. Thus, VSMC plasticity and signaling are major targets for normal and early vascular aging (9, 11).

Recently three major concepts emerged, that are discussed below in the context of arterial aging and stiffening (11, 12). Firstly, central pulse pressure, thus tensile pulsatile circumferential stress at the site of central large arteries, are major mechanical determinants of arterial wall remodeling. According to engineering principles (13), the fatiguing effect of cyclic stress is dependent on both the number of cycles (duration × frequency, i.e., age and heart rate) and the amplitude of each cycle (i.e., local pulse pressure). Repeated cyclic stress fatigues the structure of elastin that becomes fragmented; thus the lumen enlarges and arterial wall stiffens. This may be exaggerated by the slow turnover of elastin. In elderly hypertensives, large artery enlargement with aging is due to a long process of fracture of elastin fibers under the influence of long-term steady tensile stress and repeated and pulsatile tensile stress. Changes in vascular smooth muscle cells (VSMCs) phenotypes, such as growth and apoptosis, could also be involved. In favor of this mechanism is the fact that cyclic strain is a major determinant of gene expression, phenotype and growth of VSMCs *in vitro* (14–16). Thus, cyclic stress could play a role as pulsatile load, both fatiguing the ECM and changing the signaling of VSMCs. Secondly, changes in the cell-ECM interactions can be involved. Indeed, with aging, the architecture of cytoskeletal proteins and focal adhesion is disturbed, as well as the optimal organization of internal elastic lamellae and adventitial network. The connection of VSMCs to ECM in the media through elastin receptors is progressively lost (17). Thirdly, the permanent cross-talk between large and small arteries acts as an amplifier of target organ damage in elderly hypertensives since on the one side large artery stiffening gives rise to hyperpulsatility at the level of small arteries, and on the other side small arteries are damaged by hyperpulsatility.

## Consequence of Arterial Stiffness on Pressure Pulsatility

**Figure 1:**
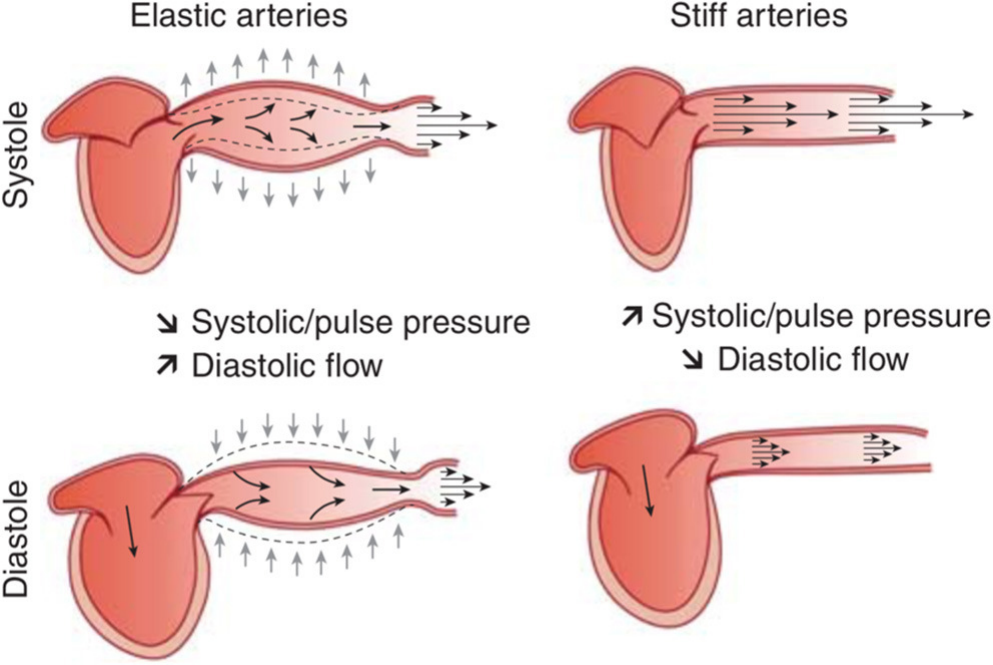
Schematic representation of the role of arterial compliance (i.e., the inverse of arterial stiffness) in dampening blood pressure pulsatility and assuring adapted blood flow through the peripheral circulation. From Briet et al. (20) with permission.

The wording “arterial stiffness” is a general term that refers to the loss of arterial compliance and/or changes in vessel wall properties (1, 18). The classical view is that compliance of large arteries, including the thoracic aorta that has the major role, represents their ability to dampen the pulsatility of ventricular ejection and to transform a pulsatile pressure (and flow) at the site of the ascending aorta into a continuous pressure (and flow) downstream at the site of arterioles. This allows to lower the energy expenditure during organ perfusion and to protect small arteries of target organs (mainly the brain and the kidney) from the damaging effects of pressure pulsatility (19). Indeed, during ventricular contraction, part of the stroke volume is forwarded directly to the peripheral tissues and part of it is momentarily stored in the aorta and central arteries stretching the arterial walls and raising local blood pressure. A more contemporary view is that arterial compliance is a key thermodynamic optimization of cardiovascular energetics. Part of the heart energy is reoriented to the distension of the arterial wall. This energy is thus “stored” in the vessel walls during systole, and recoils the aorta during diastole. This phenomenon squeezes the accumulated blood forward into the peripheral tissues, ensuring a subsequent diastolic flow (Figure 1). The stiffness and geometry of the arteries make this phenomenon effective (19, 21). When the stiffness is low (young healthy subject), a large amount of cardiac energy is redistributed during diastole and helps decreasing post-load and improving organ perfusion during diastole (especially “torrential” circulations such as brain, kidney, and coronary arteries). In elderly hypertensives, a higher pressure is necessary to stretch a more rigid arterial system. Thus, this is mainly during systole that a larger proportion of the stroke volume flows through the arterial system and peripheral tissues. The main consequences are an intermittent flow and pressure, an exaggerated flow and pressure pulsatility at the site of distal small resistance, and a shorter capillary transit time. The later reduces metabolic exchanges. Altogether, these mechanisms damage target organs.

## Arterial Stiffness and Wave Reflection in the Elderly

The pulsatility of blood pressure is exaggerated in the elderly by the phenomenon of wave reflection (19, 21). With aging and hypertension, the large arteries stiffen and pulse pressure (PP = systolic *minus* diastolic) increases at the site of central and peripheral arteries. Because rhe arterial tree can be approximated to a viscoelastic tube with numerous branches and a high level of impedance (resistance in oscillatory conditions) of the tube end, retrograde waves, due to wave reflection, are generated. The higher the arterial stiffness, the higher the transmission velocity of both forward and reflected waves, thus the reflected wave arrives earlier in the central aorta and augments pressure in late systole, increasing central PP (19, 22).

**Figure 2:**
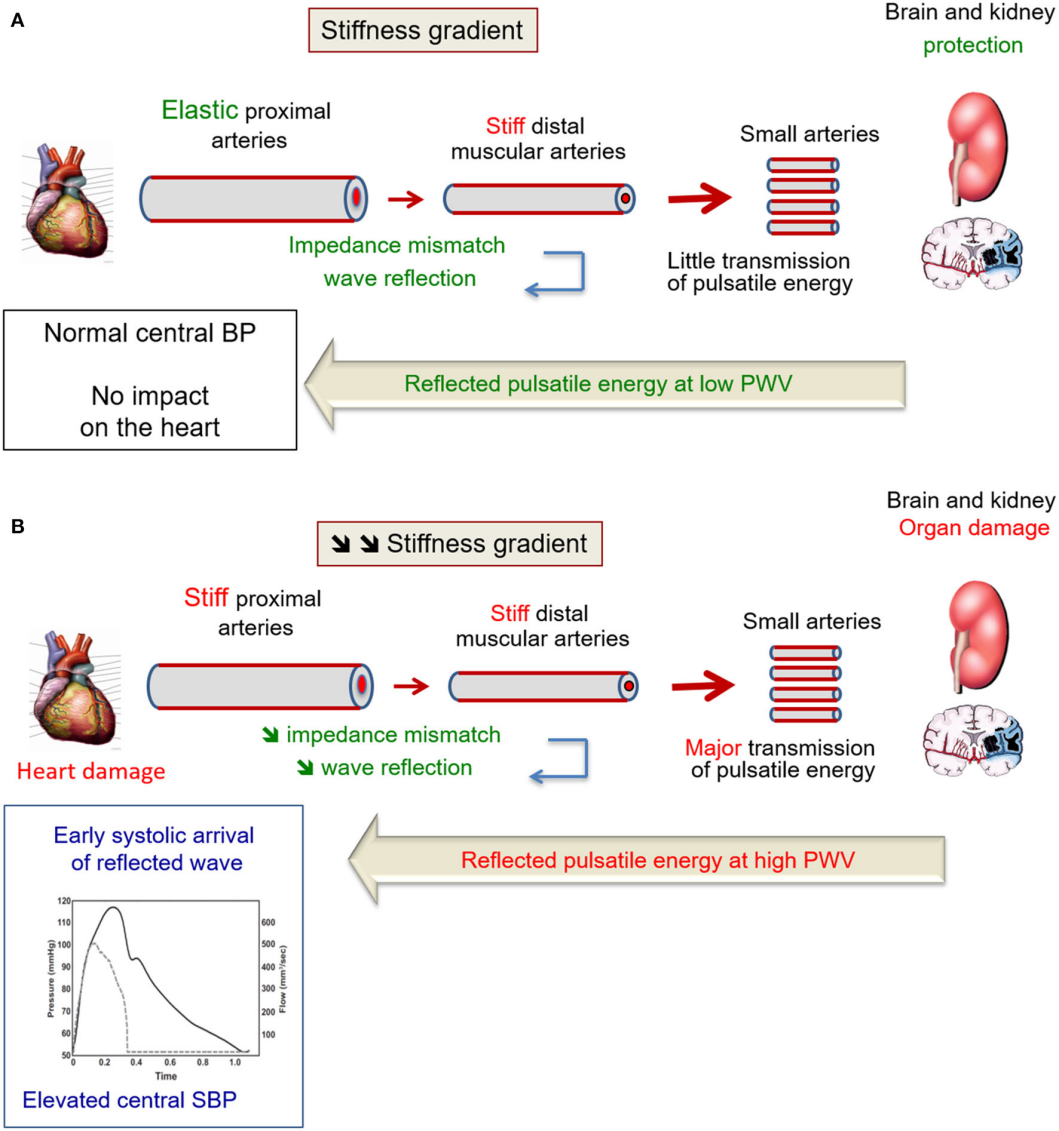
**(A)** Healthy aging is a means to retard brain, kidney, and heart damage. Indeed, in healthy subjects, an impedance mismatch occurs in response to the stiffness gradient between proximal elastic arteries and distal muscular arteries. This phenomenon generates pressure wave reflection, limiting the transmission of pressure pulsatility to target organs. The largest part of the reflected pulsatile energy that propagates backward, toward the heart, travels indeed at low velocity along elastic arteries, thus do not superimpose on incident pressure wave. Thus, central BP remains normal. From Laurent and Cunha (23) with permission. **(B)** Wave reflections in the elderly. When the aorta stiffens with aging, it loses its ability to dampen the pulsatility of ventricular ejection. Small arteries of target organ are damaged by the hyperpulsatility. Because the stiffness of distal muscular arteries does not change with age, there is a reduction of the stiffness gradient between proximal elastic arteries and distal muscular arteries. Thus, pressure pulsatility is transmitted to a larger extent toward small arteries of target organs. The largest part of the reflected pulsatile energy that propagates backward, toward the heart, travels at high velocity along stiff arteries. TRhus, it superimposes on incident pressure wave and increase central SBP. From Laurent and Cunha (23) with permission.

To better understand the mechanisms leading to wave reflection and augmented central pulse pressure in hypertension, it is important to take into account the heterogeneity of elastic properties along the arterial tree, resulting in a stiffness gradient. In young normotensive subjects, the stiffness gradient is illustrated by the increase in arterial stiffness from upstream proximal large arteries to downstream distal medium-size arteries (Figure 2A). Although all of the large artery are constituted by three layers (intima, media and adventitia), there is a difference between large proximal elastic arteries and medium-size distal muscular arteries regarding the relative amount of VSMCs and extracellular matrix (especially elastin) in their media. In humans, pulse wave velocity increases from 4 to 5 m/s in the ascending aorta to 5–6 m/s in the abdominal aorta then 8–9 m/s in the iliac and femoral arteries (19, 24). In middle-aged normotensive subjects, the cross-sectional distensibility, assessed with echotracking systems, decreases from 40 kPa^−1^.10^−3^ in the thoracic aorta (25) to 15–25 kPa^−1^.10^−3^ in the carotid (26–28) and brachial (28, 29) arteries, 10–15 kPa^−1^.10^−3^ in the common femoral artery to 5 kPa^−1^.10^−3^ in the radial artery (10, 28, 30). Indeed, both VSMCs and many elastic lamellae are present in the media of large proximal artery, whereas VSMCs prevail in the media of medium-size distal artery (19). In healthy subjects, the stiffness gradient between proximal elastic arteries and distal muscular arteries leads to an impedance mismatch, generating pressure wave reflection upwards that limits the transmission of pressure pulsatility downward to the small arteries of target organs (Figure 2A). The reflected pulsatile energy travels at low velocity along elastic arteries, thus do not superimpose on incident pressure wave and central SBP remains normal.

By contrasts, stiff elastic proximal arteries lose their ability to dampen the pulsatility of ventricular ejection. Thus, small arteries of target organ are damaged (Figure 2B). Because distal muscular arteries do not stiffen with age (31), the stiffness gradient between proximal elastic arteries and distal muscular arteries is reduced (and sometime inverted), and there is more transmission of pressure pulsatility toward small arteries of target organs. Most of the reflected pulsatile energy that return to the heart travels at high velocity along stiff arteries, and superimposes on incident pressure wave, thus increasing central SBP and PP. In parallel, the pulsatile pressure is not sufficiently attenuated and is transmitted downwards, damaging the microcirculation.

From this reflection phenomenon, we can understand why central systolic and pulse pressures are elevated in the elderly hypertensive. The main explanation resides in the “amplification phenomenon.” The “central-to-peripheral amplification” means that central SBP increases from the central artery compartment to the peripheral arteries. Indeed, under resting conditions in healthy humans, brachial SBP is in average 10% higher than aortic SBP, sometimes up to 30%. In the presence of physiological arterial stiffness gradient (aortic PWV lower than peripheral PWV), reflection sites are closer to peripheral sites and reflection superimposes on forward wave, leading to amplification of pulse pressure. Because PWV is low, reflection comes late, in late-systole or early diastole in central arteries, limiting pulse pressure. By contrast, when the stiffness gradient disappears or is inverted (aortic PWV higher than peripheral PWV), apparent reflection sites are much closer to central arteries, and pulsatile pressure is not sufficiently dampened at the central level, and the central-to-peripheral pressure amplification is attenuated (32).

**Figure 3:**
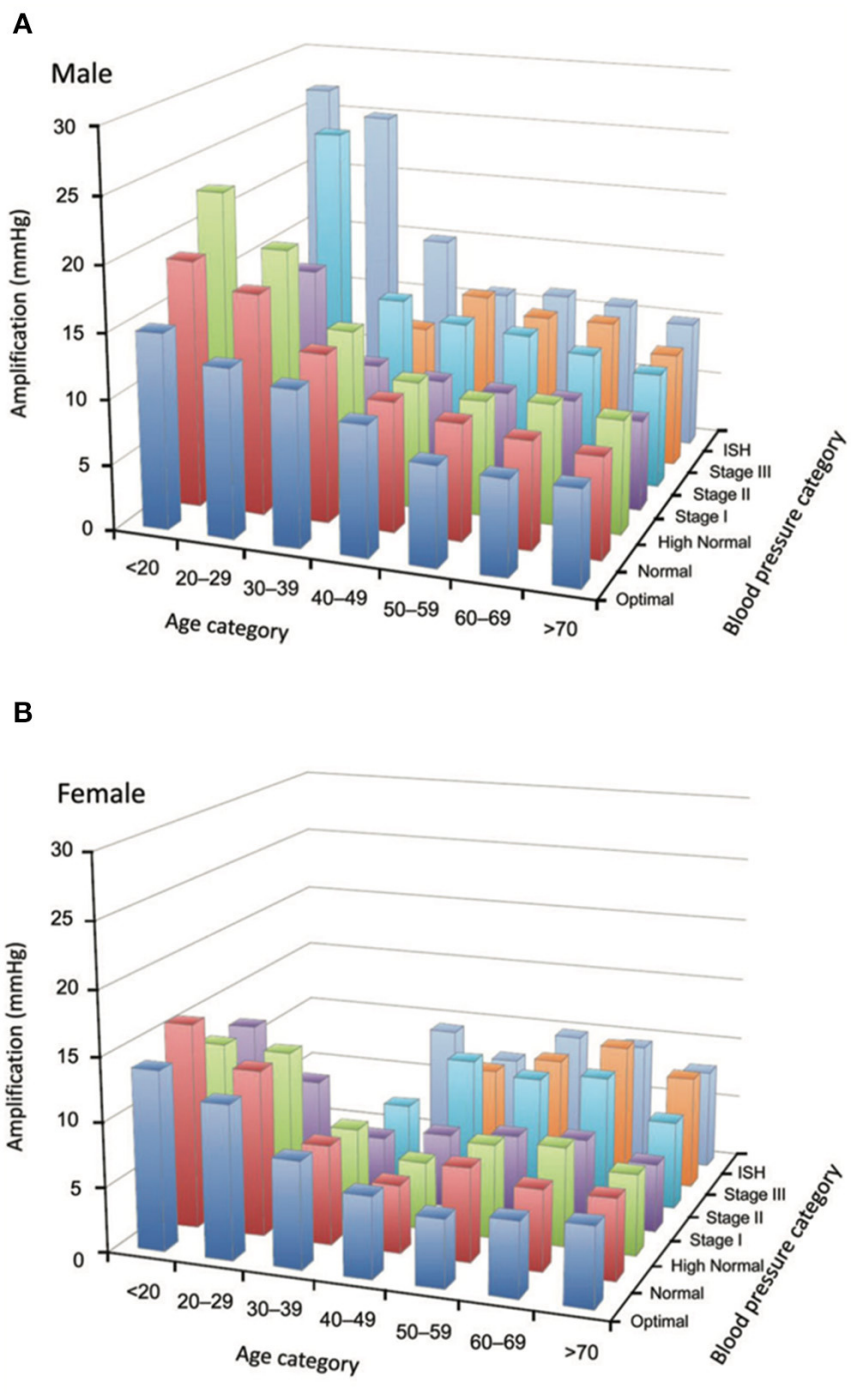
**(A,B)** Loss of amplification phenomenon in the elderly. Tridimensional bar-graphs representing amplification according to sex (6a, males; 6b, females), age categories, and blood pressure categories. The value represented here is the median of the group. Some categories are not represented because there were <50 observations. From Herbert et al. (32) with permission.

**Figure 4:**
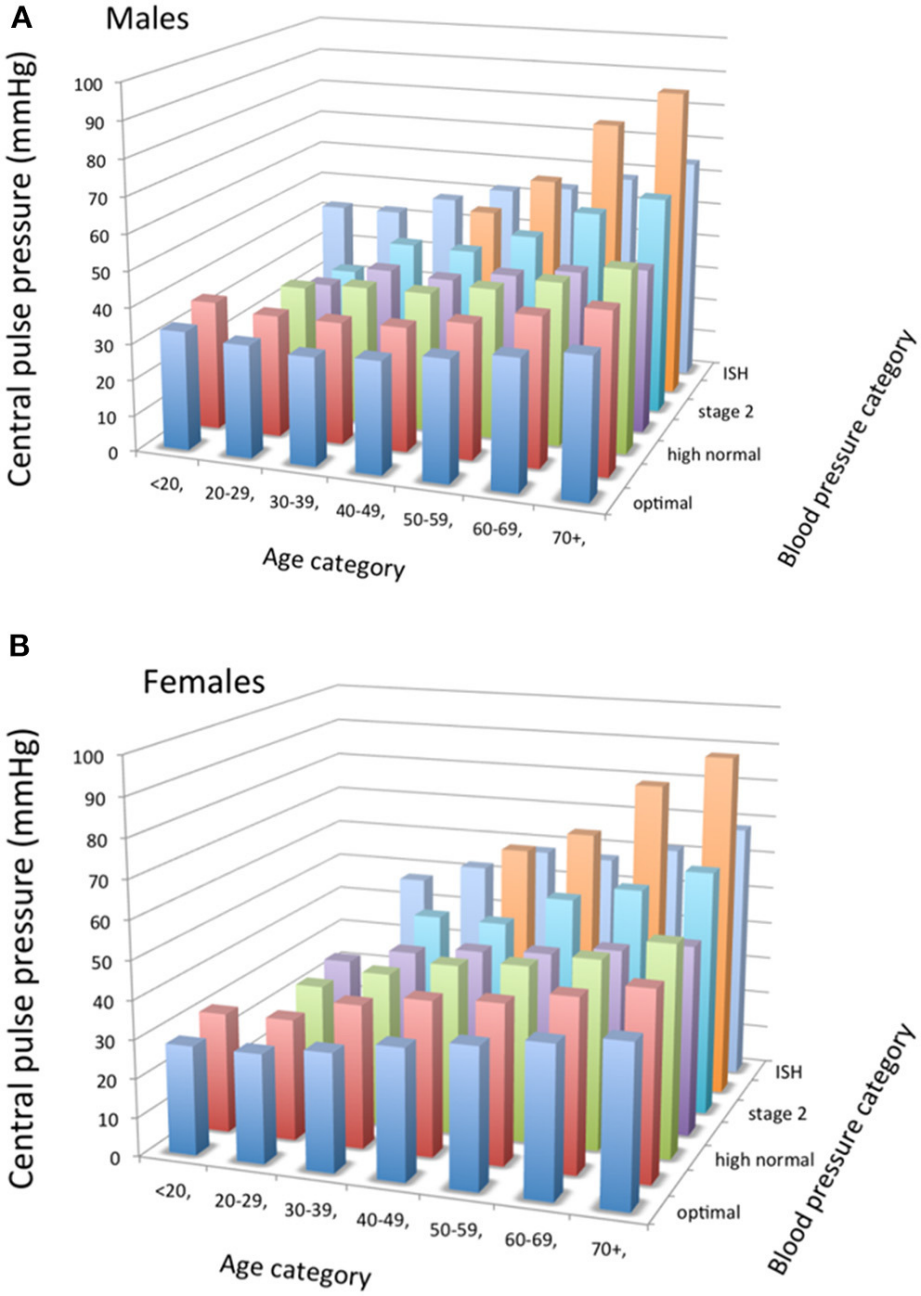
**(A,B)** Tridimensional bar-graphs representing central pulse pressure (peripheral *minus* central systolic blood pressures) according to sex (7a, males; 7b, females), age categories, and blood pressure categories. The value represented here is the median of the group. Some categories are not represented because there were <50 observations. From Herbert et al. (32), with permission.

The physiological amplification phenomenon is attenuated by aging because of arterial stiffening. Indeed, by favoring early wave reflections, arterial stiffening increases peak- and end-systolic pressures in the ascending aorta. Thus, central systolic and pulse pressures are higher in elderly subjects than in young subjects and closer to the brachial SBP value, reducing the difference (32) (Figures 3A,B, 4A,B) Excessive amplification leads to high SBP/PP in peripheral arteries, so called spurious systolic hypertension, often in young males, with various interpretations (32).

## Peripheral and Central BP in the Elderly

Cross-sectional and longitudinal population studies have shown that both peripheral SBP and DBP trajectories increase progressively between adolescence and adulthood (33, 34). Before 50 years of age, the increase in both brachial DBP and SBP can be explained by the increase in peripheral vascular resistance. DBP plateaus around age 50 years, and then decreases. By contrast, SBP continues to rise even after the age of 50 in response to the continuous age-induced increase in arterial stiffness, as explained above. After age 60, the divergent trajectories of SBP and DBP explains why PP begins to increase after age 50. The rapid widening of PP is considered as caused by arterial stiffening.

For these reasons, isolated systolic hypertension (ISH), defined as SBP > 140 and DBP <90 mmHg, represents the most frequent subtype of hypertension after age 60 (34). Men and women have different trajectories. The prevalence of hypertension in young women is lower than in men of the same age. And then, BP in women reaches men's values around the fourth decade of life. By the seventh decade, BP in women is higher than in men. Since hypertension is more frequent in the elderly, and elderly women have higher BP than men, it is not surprising that 60% of all hypertensive individuals now are women (34).

Reference values for central SBP have been reported according to sex, age and BP categories (32). According to age, central SBP was higher in men than in women until age 50–59, after which the tendency was reversed. According to BP categories, central SBP was lower in men than in women within each peripheral BP category, except “optimal.” Amplification between central and peripheral BP, calculated as peripheral SBP minus central SBP, was higher in males than in females at any given age or BP value (32). The difference between sexes becomes smaller with increasing age and values of BP. Amplification is very little influenced by BP categories. Amplification decreases gradually with aging without clearly reaching a plateau, except in those with optimal brachial BP (32) (Figures 3A,B, 4A,B).

## High Central Systolic and Pulse Pressures, Target Organ Damage, and Cv Events

The small/large artery cross-talk has a synergistic effect on target organs, in large part through a high pulsatile energy that is delivered as central systolic or pulse pressure. There is a large amount of evidence that central systolic and pulse pressures are the most deleterious elements of the blood pressure load on target organs. Their increase is better correlated with hypertensive target organ damage than either brachial systolic and pulse pressures or mean BP (22, 35, 36).

### Cardiac Damage

A higher correlation has been observed with central SBP and PP than with brachial SBP and PP for left ventricular hypertrophy (37), systolic dysfunction (38), diastolic dysfunction (39). This is also the case for new onset of atrial fibrillation and left atria enlargement (40). Central SBP increases the load on the left ventricle thus the myocardial oxygen demand. In addition, arterial stiffness is correlated to left ventricular hypertrophy (LVH) (37), a known risk factor for coronary events (41). High central PP and low diastolic BP can cause subendocardial ischemia. Indeed, a high central pulsatility reduces large epicardial coronary vascular tree perfusion during diastole, thus decreases coronary flow reserve because of lesser blood pressure during early diastole (42). Rarefaction and remodeling of intramyocardial coronary artery, as well as left ventricular hypertrophy and left ventricular diastolic dysfunction also contribute to a reduction in microcirculatory flow reserve, an impairment of tissue perfusion and a higher susceptibility to ischemia during high levels of metabolic and oxygen demand (42). Atherosclerosis at the site of epicardial coronary arteries increases the damaging effects of above damages and the risk of ischemic heart disease.

### Brain Damage

The damage of large and small arteries can increase the risk of ischemic stroke, and, likely in parallel, white matter lesions, lacunar infarcts, and cognitive decline (43, 44). High pulse pressure can be transmitted into cerebral arteries, thus leading to an inward remodeling reducing lumen diameter aiming at protecting the microcirculation from pulsatile stress. The cerebral (as well as renal) circulation is particularly susceptible to pressure damage, since this is a high flow/low resistance circulation. Under such condition, mean and pulse pressures are easily transmitted from the aorta to small cerebral (and renal) arteries (45). An increased arterial pulsatility due to large artery stiffening can thus be transmitted to cerebral small vessels and associated with white matter lesions (46, 47). Indeed, the pulsatility of the carotid flow, measured with Doppler, and pressure, and the carotid-femoral pulse wave velocity, are related with silent subcortical infarcts or white matter lesions (46, 47), and lower scores in various cognitive domains (47). There is also a relationship between carotid stiffness and large white matter hyperintensity volume, independently of vascular risk factors and carotid plaque (43), and with stroke (48).

Several mechanisms can explain why an increased arterial stiffness can increase the risk of stroke: an elevated central PP, remodeling extracranial and intracranial arteries, in association with an increased carotid wall thickness and the development of stenosis and plaques (12) and the prevalence and severity of cerebral white matter lesions (43, 49). Aortic stiffening may also express damages at the site of the cerebral vasculature. Thus, it is not surprising that aortic stiffness can to predict not only incident stroke (50), but also the functional outcome after stroke, independently of classical cardiovascular risk factors (51). Another explanation is given by the differential physiological behavior of brain small arteries compared with other systemic vascular beds (22). In elderly hypertensives, the inward remodeling of small cerebral arteries and associated increased myogenic tone impairs vasomotor reactivity, limits the autoregulation of cerebral blood flow, and increases susceptibility to focal ischemia when blood pressure is transiently and/or acutely low (52). Patients with exaggerated visit-to-visit variability of blood pressure, namely SBP, are at increased risk of stroke (53), which suggests that repeated episodes of hypoperfusion and microvascular ischemia resulting from excessive variability coupled with reduced autoregulation, could favor tissue damage and stroke. Similar findings can result from exaggerated short-term BP variability, detected with ambulatory blood pressure monitoring, that is associated with arterial stiffness (54). Finally, a high central PP may remodel the arterial system not only at the site of the intracranial arteries, but also in extracranial arteries, thickening the carotid wall, leading to the development of atherosclerotic plaques (55, 56).

### Renal Damage

Myogenic tone in the renal circulation is impaired in the elderly hypertensive. The consequence is a loss of the autoregulation capacity and an increase in barotrauma due to high systolic BP, leading to glomerular injury. Because of its torrential nature (very low resistances), even small increases in peripheral blood pressure are transformed into high pulsatile energy that is transmitted to the kidney and dissipated in the microcirculation, leading to hyperfiltration and glomerulosclerosis. Clinical data are consistent with this pathophysiological approach. Significant relationships have been demonstrated between brachial pulse pressure and either glomerular filtration rate (GFR) or microalbuminuria (57); between arterial stiffness and either GFR (58, 59) or urinary albumin (59); between carotid stiffness and GFR (59); and between central PP and incident end-stage renal disease (60). Although confounding factors may not be fully excluded, there is a large amount of evidence for linking the pulsatility of BP to renal damage.

### Cardiovascular and Renal Outcome

Because the small/large artery cross-talk exerts a synergistic damaging effect on target organs, it is not surprising that arterial stiffness (2, 3, 61–64), central systolic and pulse pressures (2, 3, 18, 60), and media-to-lumen ratio of small resistance arteries (65), have independent predictive value for CV events and renal complications in hypertensive patients. Several reviews (35, 66) have already addressed this issue.

## Early Vascular Aging and Supernormal Vascular Aging in the Elderly

**Figure 5:**
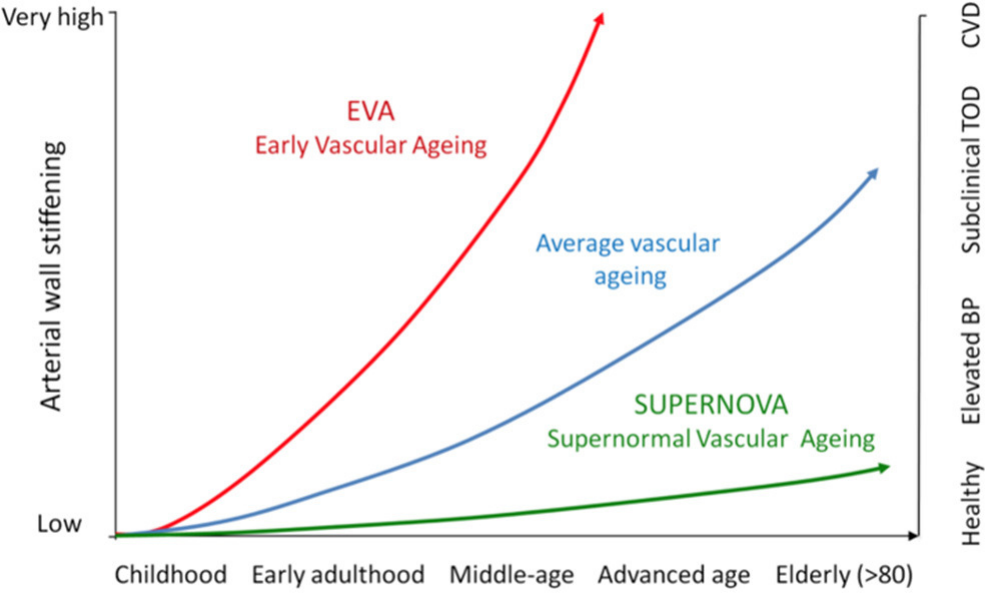
This figure illustrates, in a life-course approach of hypertension, our hypothesis that progressive arterial wall stiffening with aging parallels incident hypertension, and then subclinical target organ damage, and then CV complications. EVA subjects reach each of these steps earlier than the average population, whereas SUPERNOVA subjects remain protected for a long period of time.

Ten years ago, we promoted the concept of early vascular aging (EVA) (6, 67–69) to show that it is possible to early identify subjects with arterial damage that, if undetected otherwise, would lead them into premature cardiovascular disease and irrecoverable residual risk despite later therapeutic interventions. *The Lancet Commission on Hypertension* (70) used a similar life-course approach to better illustrate that preventive efforts should be focused on several avoidable thresholds (elevated BP, subclinical organ damage and CV events), with the goal to improve life-course trajectory as much as possible. Figure 5 illustrates the life-course concept applied to arterial stiffening. Our hypothesis is that progressive arterial wall stiffening with aging parallels incident hypertension, and then subclinical target organ damage, and then CV complications in a steeper way for some individuals (EVA) than in others (SUPERNOVA). EVA subjects reach each of these steps earlier than the average population. Thus, elderly patients with hypertension will experience target organ damage and CV events earlier than elderly subjects without hypertension or less CV risk factors.

EVA can be diagnosed in patients who present an abnormally high arterial stiffness for their age and sex. Thus, EVA represents an altered capacity for repairing arterial damage in response to aggressors like mechanical stress and metabolic/chemical/oxidative stresses. In other words, arterial stiffness is an integrator of all damages done to the arterial wall. Moreover, aortic stiffness as a marker of arterial wall damage or arteriosclerosis, integrates both the effect of risk factors and susceptibility to those risk factors and duration of exposure. Thus, arterial stiffness measures not only the current arterial damage (a product of age, risk factors and intrinsic susceptibility to them) but also its regression (when a therapeutic action is taken) or progression (when exposure continues or therapeutic actions fail). It differs from the usual “snapshot” that physicians get from their patients when they only measure BP, cholesterol and glycemia. This is why EVA-arterial stiffness has a higher predictive value for CV events than classical CV risk scores (62).

Recent cross-sectional and longitudinal studies have extended the list of the epidemiological determinants of arterial stiffness. Most of these determinants belong to the classical risk factors, either non-modifiable such as ethnicity, sex, chronological age, family history and personal history, or modifiable such as blood pressure, diabetes mellitus, dyslipidemia, and smoking (1). Additional studies underlined the role of hyperglycemia, metabolic syndrome, insulin resistance, obesity, abdominal fat, chronic kidney disease, high salt intake, chronic low-grade inflammation, oxidative stress, inadequate diet, alcohol consumption, social deprivation, perceived stress, and a number of genetic factors (1, 7).

More recently, we extended the concept of extremes in vascular aging (7). Indeed, values of arterial stiffness are scattered at a given age for any level or category of risk factors. If attention has been focused on subjects with high arterial stiffness (EVA), low and very low values have drawn little attention. We proposed that very low values of arterial stiffness, whatever the level of risk factors, define a protective phenotype and we proposed to call this phenotype super-normal vascular aging or SUPERNOVA (7). The values of arterial stiffness in SUPERNOVA subjects are by definition lower than the values of arterial stiffness in the average population and even lower than in healthy subjects. The main concept is that SUPERNOVA subjects are protected against the influence of CV risk factors, despite being exposed to them. The difference with EVA is that, at a given age, CV risk factors are not translated into subclinical organ damage and CV complications. Thus, a minority of elderly hypertensive may have lower values of arterial stiffness than expected for their age and hypertensive condition. This possibility should be considered when taking in charge elderly hypertensive patients, and this is why we recommend that the measurement of arterial stiffness should be performed as a routine check-up.

## Clinical Measurement of Arterial Stiffness

Arterial stiffness can be evaluated at the systemic, regional and local levels. Systemic arterial stiffness can only be estimated from models of the circulation. By contrast, regional and local arterial stiffness can be measured directly, and non-invasively, at various sites along the arterial tree. A major advantage of their evaluation is that they are based on direct measurements of parameters strongly linked to wall stiffness. A large number of reviews have been published on methodological aspects (1, 71, 72). We will only discuss here the measurement of regional stiffness, because it is used most often and it is recommended by international guidelines (73). Local determination of arterial stiffness, obtained either with the well-established high-resolution echotracking systems or more recently with magnetic resonance imaging are rather indicated for pathophysiological and pharmacological studies (1, 71, 72).

**Figure 6:**
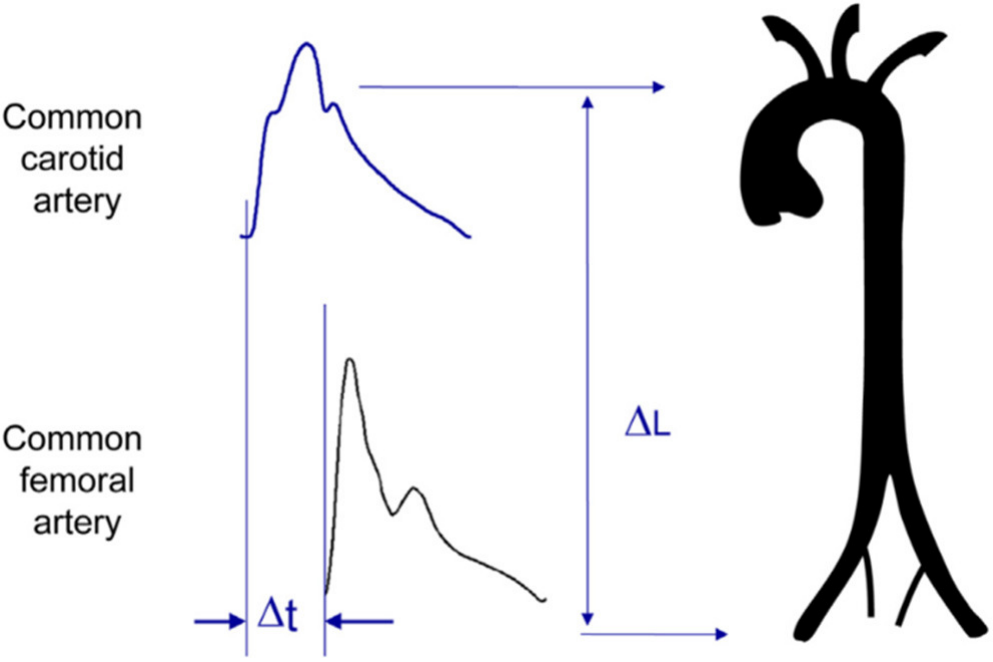
Measurement of carotid-femoral pulse wave velocity with the foot-to-foot method. From Laurent et al. (1), with permission. The waveforms are usually obtained transcutaneously at the right common carotid artery and the right femoral artery. The time delay (Δt, or transit time) is measured between the feet of the two waveforms. The distance (ΔL) covered by the waves is usually assimilated to the surface distance between the two recording sites, i.e., the common carotid artery and the common femoral artery. PWV is calculated as PWV = 0.8 × ΔL (meters)/Δt (seconds).

Regional stiffness is mainly determined through pulse wave velocity between two arterial sites. The measurement of pulse wave velocity (PWV) is generally accepted as the most simple, non-invasive, robust, and reproducible method with which to determine regional arterial stiffness (1, 5). Carotid-femoral PWV (cfPWV) is most often directly measured along the aortic and aorto-iliac pathway. This is the most clinically relevant measurement, since the aorta is responsible for most of the pathophysiological effects of arterial stiffness. Carotid-femoral PWV is measured using the foot-to-foot velocity method, and calculated as cfPWV = D (meters) /Δt (seconds), where D is the distance covered by the waves, usually assimilated to the surface distance between the two recording sites from various waveforms [corrected by a factor 0.8 to account for arterial path (5)], and Δt is the time delay (or transit time) (Figure 6). Since the first reports by Bramwell and Hill (74), cfPWV methods have improved by using high precision tonometers and computing. This method has been extensively validated, standardized and referenced (1, 5, 72). A large number of studies (50, 61, 62, 75), including a collaborative study (76) in 16,867 subjects and patients, showed that age and BP were the main determinants of cfPWV, and that at a given age, cfPWV was higher in hypertensives than in normotensives (Table 1).

**Table 1:** Distribution of carotid-femoral pulse wave velocity (cfPWV, m/s) according to the age category in the normal values population (1,455 subjects).

**Age category (years)**	**Mean (±2 SD)**	**Median (10–90 pc)**
<30	6.2 (4.7–7.6)	6.1 (5.3–7.1)
30–39	6.5 (3.8–9.2)	6.4 (5.2–8.0)
40–49	7.2 (4.6–9.8)	6.9 (5.9–8.6)
50–59	8.3 (4.5–12.1)	8.1 (6.3–10.0)
60–69	10.3 (5.5–15.0)	9.7 (7.9–13.1)
>70	10.9 (5.5–16.3)	10.6 (8.0–14.6)

*SD, standard deviation; pc, percentile*.

*From Mattace-Raso et al. (76), with permission*.

Alternative methods have been proposed by measuring transit time between the arm and the leg (brachial-ankle PWV), the heart and the leg (heart-ankle PWV), or between the finger and the toe (finger-toe PWV). Particularly, the measurement of brachial-ankle PWV - baPWV (Omron, Japan) has been developed as an automatic cuff-based method in order to increase easiness and acceptability (77). Brachial and post-tibial arterial pressure waveforms are simultaneously detected by cuffs connected to a plethysmographic sensor and an oscillometric pressure sensor wrapped on both arms and ankles. The traveled distance is automatically calculated based on patient's height. Transit time is the time delay between the proximal and distal “foot waveforms.” These alternative techniques are simpler, at the cost of imprecisions and ambiguities on the arterial path (72). Methods using a single-site cuff-based pulse wave velocity measurement are promising but await epidemiological validation.

## Clinical Measurement of Central Pressure and Wave Reflexion

Several reviews have made recommendations for adequate measurements of central BP (1, 78–82). Arterial pressure waveform should practically be analyzed at the central level, i.e., the ascending aorta, since it represents the true load imposed to the heart, the brain, and the kidney, and more generally to central large artery walls. The pressure waveform is generally measured non-invasively with a pencil-type probe incorporating a high-fidelity Millar strain gauge transducer (SPT-301, Millar Instruments). A common approach is to perform radial artery tonometry and then use a transfer function (Sphygmocor, AtCor, Sydney Australia) to calculate the aortic pressure waveform from the radial waveform (83, 84). Indeed, the radial artery is well-supported by bony tissue, making optimal applanation easier to achieve.

Aortic pressure waveform can also be deduced from the common carotid artery waveform. Carotid tonometry requires a higher level of technical expertise, but a transfer function is not necessary since the arterial sites are very close and waveforms are similar (1). In addition to methods determining the pressure waveform at the central site, novel methods have been developed. Their goal is to determine the discrete value of central SBP using the second systolic peak (SBP2) on the radial or brachial pressure waveforms (1, 78, 79, 81, 82). Wave separation analysis allows deriving central pressure forward, pressure backward and reflection magnitude. This is obtained by using a generalized transfer function on the radial artery pressure, in order to obtain the central pressure waveform (85).

## Predictive Value of Arterial Stiffness and Wave Reflection

The measurement of arterial stiffness and wave reflections in clinical practice in hypertensive patients is comforted by the repeated demonstration that arterial stiffness has an independent predictive value for CV events (1, 5).

Several longitudinal epidemiological studies have demonstrated the predictive value of arterial stiffness for CV events. The largest amount of evidence has been given for aortic stiffness, measured through carotid-femoral PWV. Aortic stiffness has independent predictive value for all-cause and CV mortality, fatal and non-fatal coronary events, and fatal strokes not only in patients with uncomplicated essential hypertension (50, 61, 62), but also in patients with type 2 diabetes (75) or end-stage renal disease (86), in elderly subjects (87), and in the general population (64, 88).

Two meta-analyses (2–4) consistently showed the independent predictive value of aortic stiffness, measured by carotid-femoral PWV, for fatal and non-fatal CV events in various populations, as well as two meta-analyses on carotid stiffness (48, 89). The measurement of carotid-femoral PWV is recommended in the 2018 ESC-ESH Guidelines for the management of hypertension (73). Brachial-ankle PWV has also demonstrated a predictive value for CV events (90) and its measurement is recommended in the 2019 Japanese Society of Hypertension guidelines for the management of hypertension (91).

Aortic stiffness has demonstrated an independent predictive value for CV events after adjustment to classical CV risk factors, including brachial pulse pressure. These data show that aortic stiffness predicts CV events to a larger extent than each of classical risk factors. In addition, aortic stiffness retains its predictive value for CHD events after adjustment to the Framingham risk score, demonstrating an added value to a combination of CV risk factors (62). Aortic and carotid stiffness have additive predictive value (48). As discussed above, one reason may be that aortic stiffness is an integrator of the cumulative damage of CV risk factors on the aortic wall over a long period of time, whereas the classical CV risk factors, such as BP, glycemia, and lipids can fluctuate over time. Thus, their values, measured at the time of risk assessment, may not reflect the true values damaging the arterial wall.

**Figure 7:**
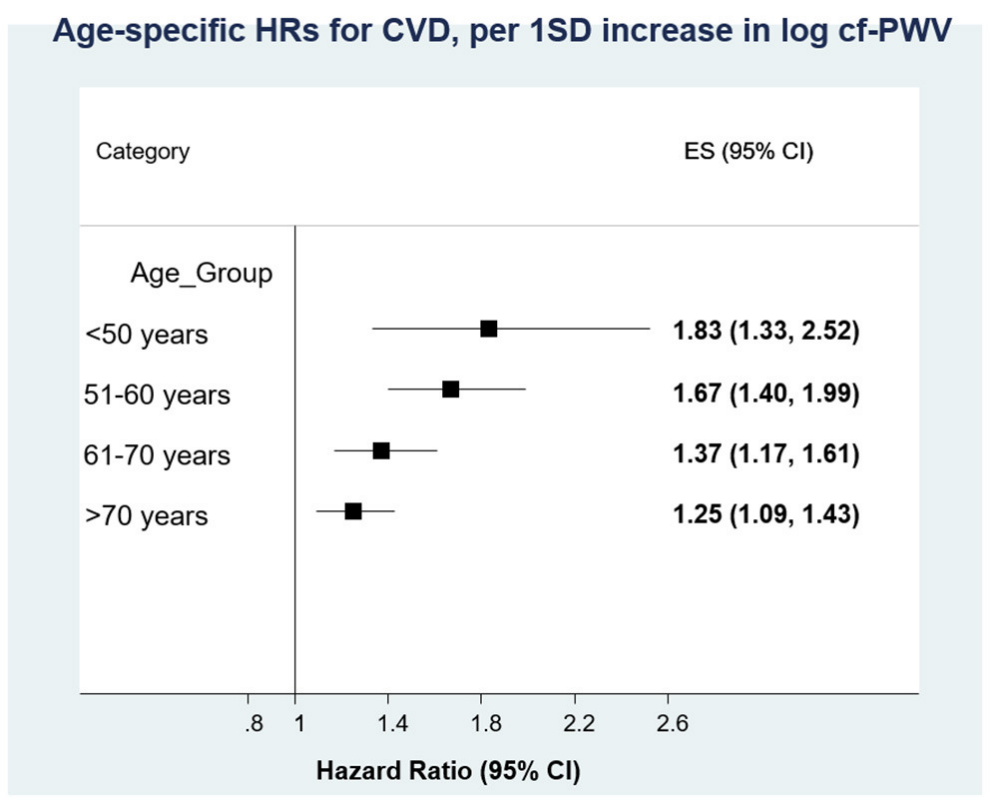
Individual participant systematic review of the predictive value of arterial stiffness (carotid-femoral pulse wave velocity) for cardiovascular disease. Associations stratified according to sex, diabetes, and hypertension were similar but decreased with age, with a significant interaction. From Ben Shlomo et al. (4), with permission.

An important issue is whether arterial stiffness retains its independent predictive value in elderly hypertensives. Ben Shlomo et al. (4) undertook a systematic review and obtained individual participant data from 16 studies gathering 17,635 participants. cfPWV demonstrated predictive value for coronary heart disease, stroke, and CVD. Associations stratified according to sex, diabetes, and hypertension were similar but decreased in intensity with age: 1.89, 1.77, 1.36, and 1.23 for age < 50, 51–60, 61–70, and >70 years, respectively, for one standard deviation, with a significant interaction (Figure 7). Thus, although the predictive value of arterial stiffness for CV events is higher in adults <50 years, it is still significant in elderly.

## Pharmacology of Arterial Stiffness and Wave Reflection

Several reviews (1, 92) and studies (31, 88, 93, 94) reported the changes in arterial stiffness and wave reflections in hypertensives after various interventions, either non-pharmacologic or pharmacologic. Non-pharmacological treatments that are able to reduce arterial stiffness include (1) weight loss, exercise training, dietary changes, low salt diet, moderate alcohol consumption, and hormone replacement therapy.

Pharmacological treatments which are able to reduce arterial stiffness in humans include (a) antihypertensive treatment (95), such as diuretics in old people, beta-blockers, ACE inhibitors (94, 96), angiotensin receptor blockers (ARBs) (31), and calcium channel antagonists (93); (b) treatments of congestive heart failure, such as angiotensin converting enzyme (ACE) inhibitors (96) and vasopeptidase inhibitors (97, 98); (c) hypolipidemic agents such as statins (99); (d) antidiabetic agents, such as thiazolidinediones (100); and (e) advanced glycation end products (AGE)-breakers, such as alagebrium (ALT-711) (101). The reduction in arterial stiffness in response to antihypertensive treatment can be only due to BP lowering, but additional BP-independent effects may be involved. However, some studies unequivocally showed that antihypertensive treatment was able to reduce arterial stiffness and/or wave reflections independently of the reduction in brachial BP. This has been observed after a calcium channel blocker (93), long term ACE inhibition (94) or angiotensin-receptor blockade (31). Moreover, certain drugs not targeting blood pressure can decrease arterial stiffness (statins, antidiabetics, anti-inflammatory drugs for instance), showing that arterial stiffness can regress even with no change in BP.

An open issue is whether, in the elderly, life-style changes and pharmacological treatments are effective in reducing arterial stiffness to the lower levels of younger subjects, and whether the reduction in arterial stiffness translates into a reduction in cardiovascular events. To our knowledge, no large randomized clinical trial has been performed in a specific group of elderly hypertensives, either above 60 or 70 years of age.

## The Particular Case of Very Old Subjects

Subjects older than 80 years represent a particular population since they are generally frail, with several comorbidities. Very few studies have been done in very elderly hypertensives. The HYVET study (102), one of the rare large randomized clinical trial performed so far in very old hypertensives (average 84 years), showed that antihypertensive treatment with indapamide, with or without perindopril, can be beneficial. This was confirmed by Corrao et al., (103), in a nested case-control study on a cohort of patients aged 85 years or older (average 88 years). These patients were newly treated with antihypertensive drugs and the level of drug adherence was available. Compared with patients with very low adherence, patients with high adherence (103) showed a significant risk reduction for death and all the outcomes combined. The risk of heart failure and stroke was also reduced, whereas the risk of MI was not affected by adherence with antihypertensive drugs. Interestingly, similar findings were obtained in the cohort of patients aged 70–84 years.

Very few studies addressed the particular case of very elderly individuals. The PARTAGE study is an observational one (104, 105), that followed 682 individuals aged older than 80 during 2 years, and showed that arterial stiffness as evaluated by carotid-femoral pulse wave velocity (cfPWV) was associated with a more pronounced cognitive decline over a 1-year period in very old frail institutionalized individuals. These data suggest that even in very old, frail individuals, arterial stiffness remains a determinant of cognitive decline, morbidity, and mortality (106).

## Conclusion

One reason for which isolated systolic hypertension represents the most frequent subtype of hypertension in the elderly is because arterial stiffness increases with aging, in some more than others, a phenomenon leading to increased wave reflection and augmented central pulse pressure. High central systolic and pulse pressures damage target organs that ultimately leads to CV and renal complications. In the elderly, arterial stiffness has predictive value for CV and renal events. Even in the very old frail hypertensive, arterial stiffness retains its predictive value for cognitive decline and morbidity-mortality. The measurement of arterial stiffness may help the physician to better determine the risk of CV complications in elderly hypertensive and adapt the therapeutic strategy.

## Author Contributions

All authors listed have made a substantial, direct and intellectual contribution to the work, and approved it for publication.

## Conflict of Interest

The authors declare that the research was conducted in the absence of any commercial or financial relationships that could be construed as a potential conflict of interest.
